# Synthesis and CO Oxidation Activity of 1D Mixed Binary Oxide CeO_2_-LaO_*x*_ Supported Gold Catalysts

**DOI:** 10.1186/s11671-017-2352-x

**Published:** 2017-11-02

**Authors:** Huanhuan Yu, Siyuan Zhong, Baolin Zhu, Weiping Huang, Shoumin Zhang

**Affiliations:** Department of Chemistry, Key Laboratory of Advanced Energy Material Chemistry (MOE), and TKL of Metal and Molecule Based Material Chemistry, Nankai University, Tianjin, 300071 People’s Republic of China

**Keywords:** Nanorods, Catalyst, CO oxidation, Activity, Stability

## Abstract

One-dimensional (1D) Ce-La nanorods with different La contents (Ce and La in the molar ratio of 1:0, 3:1, 1:1, 1:3, and 0:1) were synthesized by hydrothermal process. Au/Ce-La nanorod catalysts were obtained by a modified deposition-precipitation method. The samples were characterized by N_2_ adsorption-desorption (BET), ICP, X-ray diffraction (XRD), SEM, TEM, EDX, X-ray photoelectron spectroscopy (XPS), UV-vis diffuse reflectance spectroscopy (UV-vis DRS), and temperature-programmed reduction (H_2_-TPR). It revealed that La existed as LaO_*x*_ in the 1D nanorods. The catalysis results demonstrated that the mixed binary Ce-La nanorod oxides could be a good support for gold catalysts. The contents of La had an important influence on the catalytic performance of Au/Ce-La nanorod catalysts. Among the catalysts, when the Ce/La molar ratio was 3:1, the 1.0%Au/Ce_0.75_-La_0.25_ nanorods pretreated at 300 °C showed the best activity among the catalysts for CO oxidation, which could convert CO completely at 30 °C. The catalysts also performed high temperature resistance and good stability for CO oxidation at the reaction temperatures of 40, 70, and 200 °C.

## Background

As a very harmful gas, CO can strongly binds to the iron atom in blood hemoglobin preventing the release of oxygen. So, its presence indoors can even cause the death of human beings and animals in the short time. It has become an increasingly severe problem on air pollution. Catalytic CO oxidation has been one of the most effective solutions for CO removal to solve such serious environmental problem [1–8]. It has also received a great deal attention recently by the scientific community in the fields of the pollution control devices for vehicle exhaust purification, indoor air cleaning, and low-temperature CO sensors [6–10]. In many cases, the precious Au dispersed on specific metal oxides with high oxygen storage capacity such as CeO_2_, TiO_2_, and Fe_2_O_3_ are highly effective candidates towards the CO oxidation [11–13]. Over the past decades, studies on the supported gold catalysts for CO oxidation at low temperatures have resulted in unexpected observations. It is generally accepted that the catalytic activities of Au catalysts depend strongly on the nature of Au nanoparticles and properties of the supports, such as the gold particle size, the Au metal-support interaction and the reducibility of the support [14–18].

As one of the most important rare earth oxides, CeO_2_ has been widely used in three-way catalysts as an efficient catalyst support due to its unique physical and chemical properties [6, 8, 15, 17]. CeO_2_ has an excellent oxygen storage and release capacity due to the ability to switch Ce^4+^/Ce^3+^, which makes CeO_2_ become an active oxide component of various oxidation catalysts used in diverse redox catalytic reactions [17–32]. Surface areas, mesoporous structures, lattice defects, and synergistic effects with other dopants can all promote the catalytic properties of ceria nanomaterials [3, 22]. To further improve the performance of Au-CeO_2_ catalysts for CO oxidation reaction, many strategies have been tried, such as preparation methods including deposition-precipitation, coprecipitation, and urea-gelation coprecipitation, which has been used to control and optimize the interaction of the Au-O-Ce structure, as well as the size and shape of ceria [33–35]. Attempts have been also made by the surface modification of support [4, 5, 22, 24, 26, 36–38]. It has been found that the use of binary mixed oxides as support could provide a good solution for the stabilization of gold nanoparticles. Moreover, the promotion by noble or transition metal enhances ceria reducibility and facilitates the formation of surface oxygen vacancies. Meanwhile, doping with transition metal cations has been proved to be an effective method to promote the physicochemical properties of one-dimensional (1D) nanostructured nanomaterials, such as catalytic activity [38–40]. Wang et al. [5] modified the surface of Au/CeO_2_ with highly dispersed CoO_*x*_ and demonstrated excellent catalytic activity in low-temperature CO oxidation. Ma et al. [37] reported that CaO, NiO, ZnO, Ga_2_O_3_, Y_2_O_3_, ZrO_2_, and rare earth additives to gold-titania catalyst are beneficial for CO oxidation, and the doped catalysts could show significant activity at ambient temperature after 500 °C aging. Park et al. [38] reported that CeO_*x*_ modified TiO_2_ support is a good catalyst for water gas shift reaction. There have been lots of studies about mixed metal oxides for CO catalytic oxidation. These doped metal ions are either deposited on the surface of the support in the form of oxide particles or into lattice of the support, which could not form a separate oxide phase. The goal of this research is to prepare 1D binary Ce-La nanorods, which is non-perovskite or solid solution type mixed oxide. That is, in the 1D nanorod structure, the two metal oxides coexist combining the merits of the two compositions to maximize the synergistic effect. Due to potential technological applications, a lot of 1D nanomaterials including nanorods, nanowires, and nanotubes have been extensively investigated during the past years [2, 4, 41, 42]. These 1D nanostructured materials, especially 1D nanorod materials, have been studied as important supports or active components in the field of catalysis, optics, and electrochemistry, such as well-controlled silicon nanowires used in solar cells [42]. It has been found that the properties of 1D structure materials such as catalytic activity are often closely related to their crystal structure and shape. As a consequence, the development of 1D nanorod materials to tailor their electronic and catalytic properties proves to be intriguing and valuable.

Herein, we report a simple solvothermal strategy to prepare a series of mixed Ce-La nanorod composites. In the synthesis process, the LaO_*x*_ and CeO_2_ could grow together in one rod. The morphology of the final products was not influenced. The XRD and TEM results show that the La cations have existed in the form of LaO_*x*_. It was found that the dopant of LaO_*x*_ showed a positive effect on the activity of gold-ceria catalysts. Au/Ce_0.25_-La_0.75_ nanorods exhibited excellent catalytic activity for CO oxidation.

## Experimental

All chemicals in this paper were of analytical grade, and they were used as received without any purification.

### Support Preparation

The Ce-La nanorods were synthesized by conventional hydrothermal method. In a typical synthesis, solutions of NaOH (9 mol/L) and Ln(NO_3_)_3_ (Ln = Ce, La, 0.8 mol/L) were mixed and maintained vigorous stirring for 30 min at room temperature. The resulting suspension was poured into a Teflon-lined stainless steel autoclave. The autoclave was sealed and kept at 110 °C for 14 h and then air-cooled to room temperature. The resulting products were filtered, washed with deionized water and absolute alcohol, dried at 80 °C for 12 h, and then calcined at 400 °C in air with a heating rate of 5 °C min^−1^ before supporting gold nanoparticles. The final products with different La contents (Ce and La in the molar ratio of 1:0, 3:1, 1:1, 1:3, and 0:1) were denoted as Ce nanorods, Ce_0.75_-La_0.25_ nanorods, Ce_0.50_-La_0.50_ nanorods, Ce_0.25_-La_0.75_ nanorods, and La nanorods.

### Catalyst Preparation

A deposition-precipitation process was carried out to prepare Au/Ce-La nanorod catalysts. Briefly, the required amount Ce-La nanorods were dispersed in 100 mL deionized water, and then mixed with a certain amount 0.01 mol/L HAuCl_4_ solution. As the pH of final HAuCl_4_ solution was about 7, which was related to the basicity of the support and acidity of HAuCl_4_, pH of the solution would be not adjusted. The suspension was keeping stirring for 12 h and refluxed at 100 °C for 4 h. After the deposition-precipitation procedure, the precipitate was centrifuged, washed with water to remove Cl^−^ ions, and dried at 80 °C under air for 12 h. The concentrations of gold were expressed as percent by mass content.

### Characterization Techniques

Gold loadings of Au/Ce-La nanorod catalysts were determined by inductively coupled plasma-atomic emission spectroscopy (ICP-9000, USA Thermo Jarrell–Ash Corp). The Brunauer–Emmett–Teller (BET) surface areas of Ce-La nanorod samples were measured by nitrogen adsorption at − 196 °C using a Micromeritics Tristar II 3020 apparatus. The XRD study was carried out on a Rigaku D/Max-2500 X-ray diffractometer (*Kα* λ = 0.154 nm) in the 2*θ* range of 3–80°. Uv-visible DRS of the catalysts were collected on a UV–vis NIR spectrophotometer (JASCO Corp V–570). TEM observations and energy dispersive X-ray analysis (EDX) were obtained with a JEM-2100 transmission electron microscope operating at 200 kV. SEM data and element mapping images were obtained with a JSM-7500F scanning electron microscope operating at 15 kV. XPS were recorded to identify the chemical composition and the oxidation state of the catalysts on a Kratos Axis Ultra DLD X-ray photoelectron spectrometer using a monochromated Al *Kα* source operated at 150 W. The binding energies were calibrated using the C 1*s* peak located at 284.6 eV. Temperature-programmed reduction (H_2_–TPR) was performed on a PX200 apparatus to measure H_2_ consumption. Prior to H_2_-TPR analysis, the samples were pretreated in He flow at 300 °C for 1 h. After cooled to 50 °C, the catalyst was reduced with 10 vol% H_2_/Ar gas flow by heating up to 900 °C at a rate of 10 °C/min.

### Catalytic Activity Test

Catalytic activity evaluation was performed in a fixed-bed flow millireactor with an inner diameter of 8 mm. Prior to reaction, 200 mg of catalyst were diluted with 17.6 g chemically inert quartz sand. Subsequently, a mixture, 10% CO balanced with air was introduced into the reactor at a total flow rate of 36.3 mL min^−1^. After holding at the reaction temperature for 30 min, the gaseous products were online analyzed by CO_*x*_ analyzer (GC-508A gas chromatography). CO conversion was calculated according to the following equation:

$$ \mathrm{CO}\kern0.5em \mathrm{conversion}=\frac{\left[\mathrm{CO}\ 2\right]}{\left[\mathrm{CO}\right]+\left[\mathrm{CO}\ 2\right]}\times 100\% $$

where [CO] and [CO_2_] represent the outlet CO and CO_2_ concentration, respectively. The temperature dependence of the sample catalytic activity was recorded in the range of 30–200 °C with a ramping rate of 10 °C min^−1^.

## Results and Discussion

### Characterization of Au/Ce-La Nanorod Catalysts

#### ICP

The amounts of gold present in Au/Ce-La nanorod catalysts were determined by ICP-AES. The results shown in Table 1 revealed that the actual amount of gold in all catalysts was lower than the nominal one. According to the preparation procedure, gold should be lost during the deposition-precipitation process.

**Table 1:** Gold loading of the Au/Ce-La samples with different supports

Samples	Nominal gold loading (%)			
	0.1	0.3	0.5	1.0
	Actual gold loading (%)			
Au/Ce nanorods	/	/	/	0.82
Au/Ce_0.75_-La_0.25_ nanorods	0.10	0.24	0.31	0.88
Au/Ce_0.50_-La_0.50_ nanorods	/	/	/	0.98
Au/Ce_0.25_-La_0.75_ nanorods	/	/	/	0.70
Au/La nanorods	/	/	/	0.73

#### BET

**Fig. 1:**
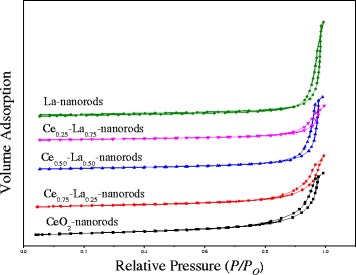
Nitrogen adsorption-desorption isotherms of Ce-La nanorods with different La content

N_2_ absorption measurements were used to measure BET surface area and average diameter on both CeO_2_ nanorods and Ce-La nanocomposites. As shown in Fig. 1, the adsorption isotherms for the Ce-La nanorods were of type IV and exhibiting characteristics of H3 hysteresis loops. All samples show a very strong increase of N_2_-adsorbed volume at a relative pressure greater than 0.85, which is a characteristic of the presence of an appreciable amount of mesoporous, [2, 22] indicating Ce-La nanocomposites comprised of aggregates (loose assemblages) forming slit-like pores. With the dopant of La, hysteresis loops shifted to a relative pressure about 0.95, which meant size of pores would become smaller, corresponding to the decrease of Ce-La composites. As presented in Table 2, specific surface area of CeO_2_ nanorods is 99.7 m^2^/g, which decreases to 74.1 m^2^/g when La is doped with the Ce/La molar ratio of 3:1. With increasing La content, surface area of Ce-La nanocomposites decreased continuously. This is mainly resulting from the content of La, which would not embed into the lattice of CeO_2_ and exist as isolate LaO_*x*_ leading to little difference in morphology of Ce-La nanocomposites. It could be observed that all nanorods have similar surface areas of 80–100 m^2^/g. Pore volume of Ce_0.75_-La_0.25_ nanorods is 0.23 cm^3^/g, which was similar to that of Ce nanorods, and larger than other Ce-La nanorods. The estimated pore diameters from BJH analysis confirmed the mesoporous nature of Ce-La nanocomposites. It is may be the advantage for the catalytic CO oxidation.

**Table 2:** BET specific surface area of the Ce-La nanorod samples with different La contents

Supports	BET surface Area (m^2^/g)	Average pore size (nm)	Pore volume (cm^3^/g)
Ce nanorods	99.7	12.4	0.31
Ce_0.75_-La_0.25_ nanorods	74.1	12.3	0.23
Ce_0.50_-La_0.50_ nanorods	62.7	13.4	0.21
Ce_0.25_-La_0.75_ nanorods	51.7	11.4	0.15
La nanorods	56.7	13.6	0.19

#### XRD

**Fig. 2:**
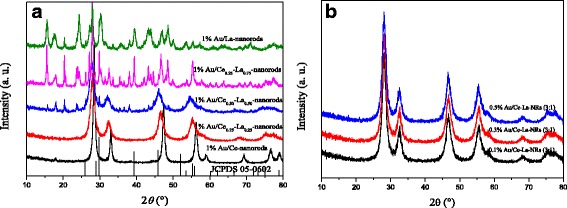
XRD patterns of 1% Au/Ce-La nanorods with different La contents (0–100 at.%) (**a**) and Au/Ce_0.75_-La_0.25_ nanorods with different Au loadings calcined at 300 °C for 2 h (**b**)

The synthesized samples were subjected to powder X-ray diffraction analysis and their structural attributes were subsequently analyzed. The crystallinity peaks for cerium oxide (Fig. 2a) were observed at 2θ = 28.6°, 33.1°, 47.6°, and 56.3° corresponding to the (111), (200), (220), and (311) diffraction planes and corroborate to the cubic fluorite structure of CeO_2_ crystal (JCPDS no. 34-0394). When the content of La was 0.25 at.%, the diffraction peaks of the La-Ce composites broaden. Peaks centered at 2θ = 30.0°, 46.0°, 52.0°, and 53.6° correspond to diffraction planes of the isolated La_2_O_3_. No peaks assigned to La(OH)_3_ could be detected. But due to the low content and approximate diffraction position, it is not easy to identify the existence of LaO_*x*_. With increasing La content, some prominent peaks are observed for La_2_O_3_ or La(OH)_3_ in the nanocomposite. The main diffraction peaks of La_2_O_3_ are present at 2θ = 30.0° (101), 39.6° (220), 46.2° (110), and 66.8° (112), which can be assigned to the hexagonal phase (JCPDS card 05-0602). The main diffraction peaks of La(OH)_3_ are present at 2θ = 15.7° (100), 27.3° (110), 27.9° (101), and 39.4° (201), which can be assigned to the hexagonal phase (JCPDS card 36-1481). The results demonstrate that La could exist as isolated La_2_O_3_ or La(OH)_3_ in the composite. After the deposition of gold, there was no diffraction peak which could be indexed to the pure face-centered crystalline structure of gold (Fig. 2b). This could be due to low content and/or small particle size of gold nanoparticles.

#### SEM and TEM

**Fig. 3:**
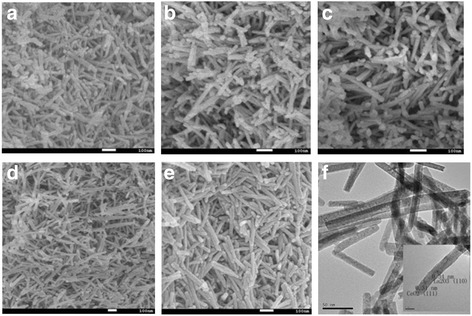
SEM images of Ce-La nanorods with different La contents: 0 (**a**), 25 at.% (**b**), 50 at.% (**c**), 75 at.% (**d**), 100 at.% (**e**); TEM image of Ce_0.50_-La_0.50_ nanorods (**f**); and the inset shows corresponding HRTEM image

Figure 3a–e shows the SEM photographs of the CeO_2_ and Ce-La nanocomposites obtained in different concentration of La^3+^ ions. It is seen that all the Ce-La nanocomposites exhibited rod-shape structure. Obviously, many rods stacked up into Ce-La bundles, leading to the formation of slit-like pores with different sizes. The results were in agreement with the N_2_ adsorption-desorption isotherms. As shown in Fig. 2a, the product mainly consists of nanorods with diameter of 5–10 nm and length of 100–300 nm. In Fig. 3e, a large quantity of nanorods with diameter of about 12.5 nm was clearly seen, and there were also a small amount of short nanorods with an average diameter of about 8.0 nm. In Fig. 3b–d, with increasing the doping concentration of La^3+^, the samples always present nanorod morphology. However, while the doping concentration was 25 mol%, the as-obtained samples displayed the most uniform nanorods with diameter of 5–20 nm and length of 100–300 nm among all the samples. Figure 3f displays TEM images of the obtained individual Ce-La nanorods. It could be seen that there are many pores in the support as revealed from the nitrogen adsorption-desorption isotherms. The HRTEM image of the Ce-La nanorods revealed that they are structurally uniform and single crystalline in nature. The lattice fringes inset in Fig. 3f illustrate two interplanar spacing values, i.e., 0.31 and 0.34 nm, which are consistent with the (111), (110) planes of the CeO_2_ and La_2_O_3_, respectively [3, 15, 43]. It revealed that the La^3+^ ions have been effectively generated into La_2_O_3_, which is consistent with the XRD spectrum.

**Fig. 4:**
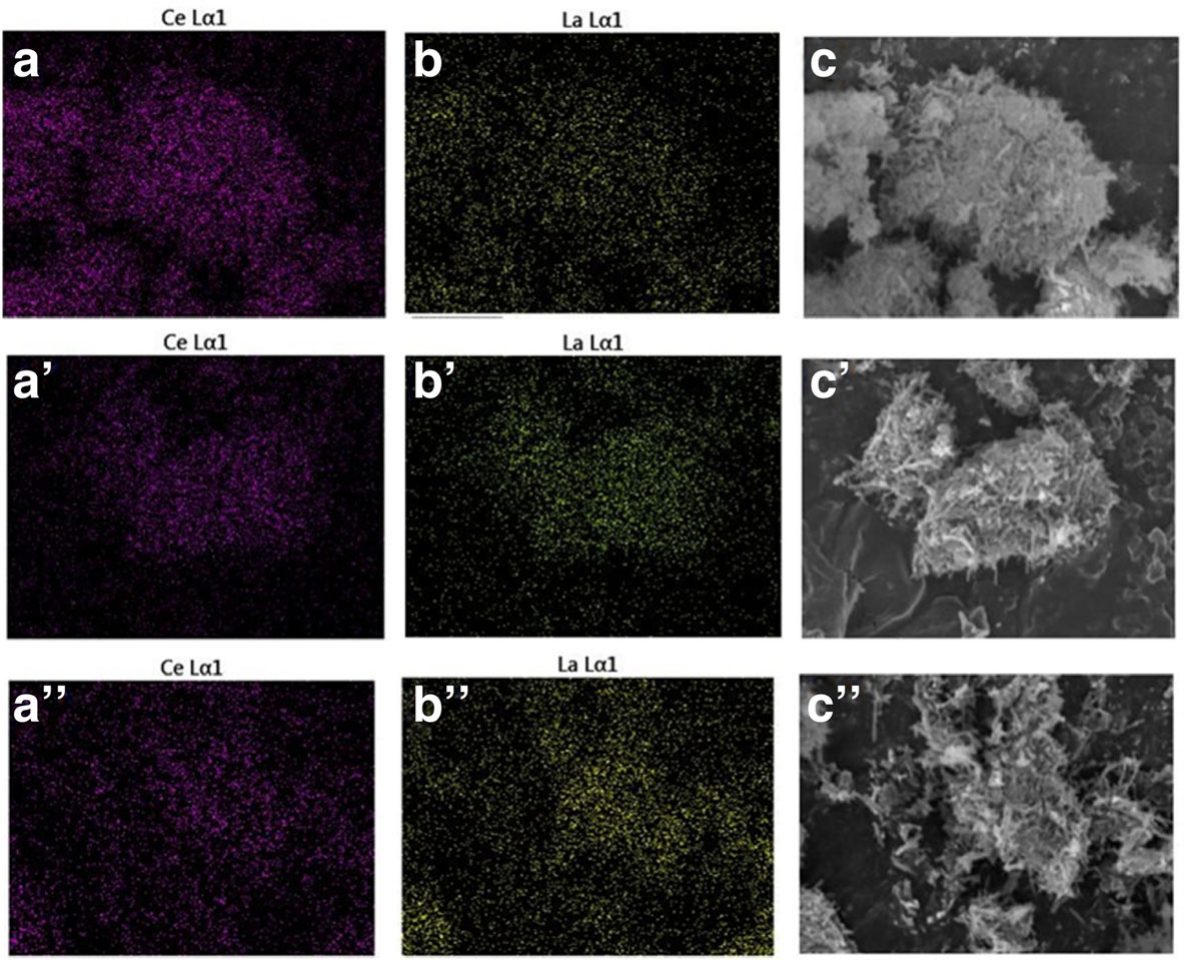
The element mapping images of Ce and La, SEM images of the mixed samples for Ce-La nanorods with La contents of 25 at.% (**a**–**c**), 50 at.% (**a**′–**c**′), 75 at.% (**a**′′–**c**″)

**Fig. 5:**
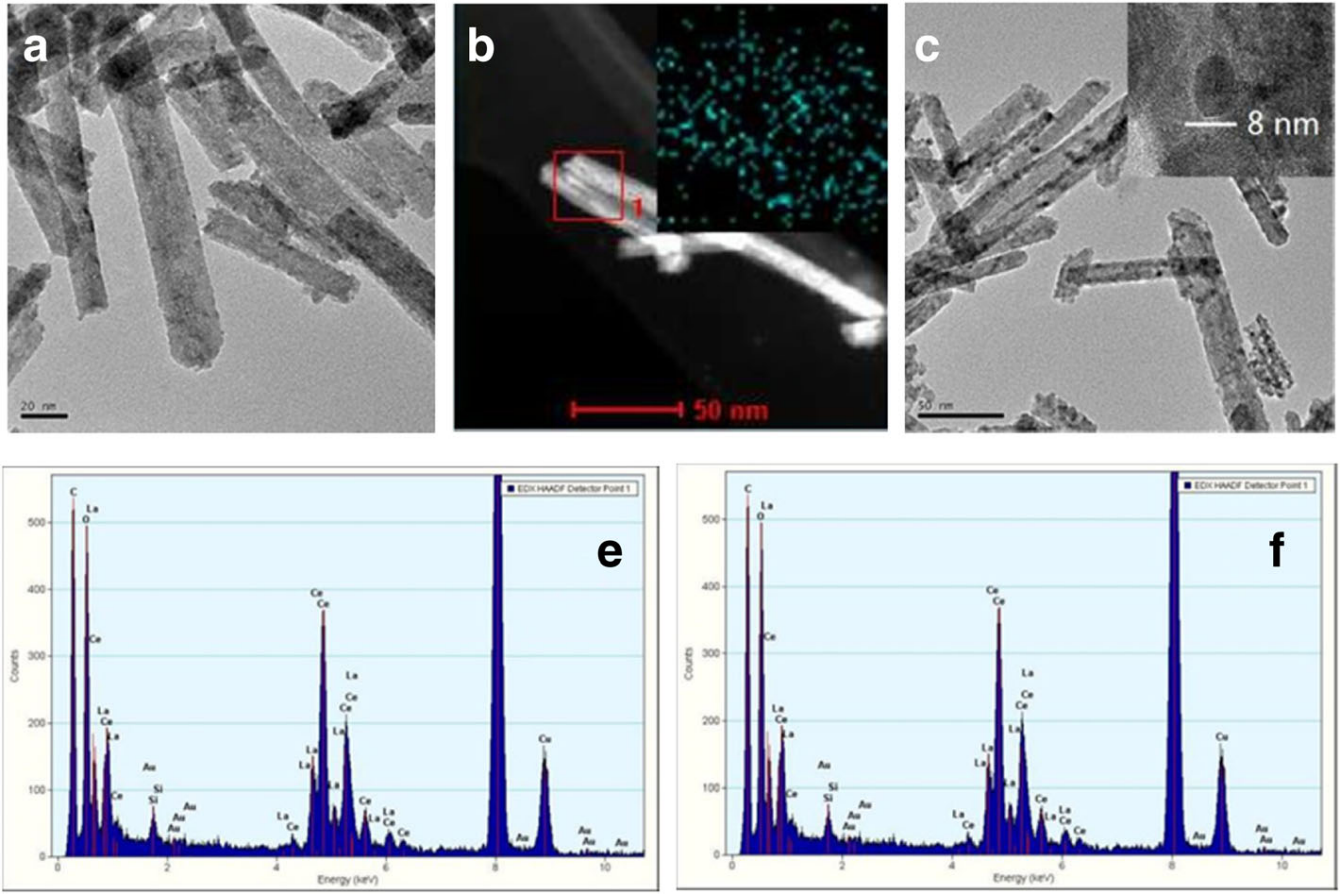
TEM and STEM images of 0.5% Au/Ce_0.75_-La_0.25_ nanorods calcined at 300 °C (**a**–**b**) and 400 °C (**c**), EDX analysis (**e**–**f**) of the images of (**a**–**b**) indicating the presence of Au signal

Element mapping and EDS analysis were employed to determine chemical composition of Ce-La samples (Fig. 4 and Table 3). The results showed uniform La/Ce molar ratios in good agreement with the expected values from the synthsis. The TEM images of Au/Ce_0.75_-La_0.25_ samples calcined at 300 °C (Fig. 5a) and 400 °C (Fig. 5c) clearly showed that the shapes of the Ce-La nanocrystals were essentially unchanged after gold addition. No gold particles were observed by TEM on the Ce-La nanorods. The presence of very highly dispersed gold clusters (*d* < 1 nm) has been evidenced by element mapping and EDX analysis (inset in Fig. 5b, e, and f). In agreement, XRD analysis performed on this sample (Fig. 2) did not reveal any peaks related to gold due to the fact that the gold particles are too small to be detected. This indicates that the Ce-La nanorod surfaces can disperse and stabilize gold atoms as sub-nanometer clusters (TEM invisible). This is in agreement with the literatures [2, 44–46]. However, some large agglomerates of gold particles (average *d* ~ 7 nm) have been observed in Au/Ce_0.75_-La_0.25_ nanorods calcined at 400 °C due to the fringes with a spacing of 0.236 nm being assigned to the (1 1 1) plane of metallic Au (Fig. 5c in which an agglomerate of gold particles is shown). It could be seen that accompanying with the high calcination temperature, gold particles obviously grew, correspondingly leading to the loss of catalytic activity.

**Table 3:** EDS results of Ce-La nanorods with different La contents

Support	wt.% (element)		at.% (element)	
	Ce	La	Ce	La
Ce nanorods	100	0	100	0
Ce_0.75_La_0.25_ nanorods	75.02	24.98	74.88	25.12
Ce_0.50_La_050_ nanorods	48.45	51.55	48.28	51.72
Ce_0.25_La_0.75_ nanorods	22.95	77.05	22.82	77.18
La nanorods	0	100	0	100

#### XPS

**Fig. 6:**
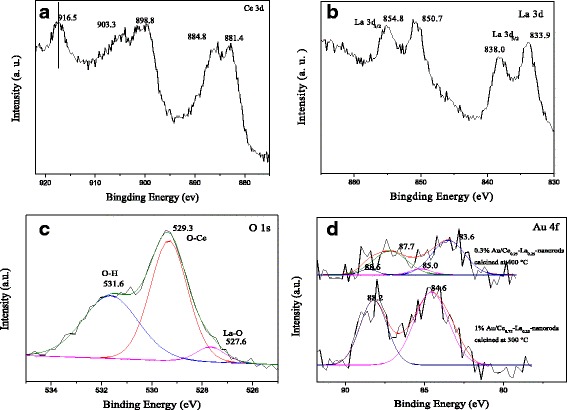
XPS spectra of 1%Au/Ce_0.75_-La_0.25_ nanorods calcined at 300 °C for 2 h: Ce 3*d* peaks (**a**), La 3*d* peaks (**b**), and O 1*s* peaks (**c**). Au 4*f* peaks (**d**) of Au/Ce_0.75_-La_0.25_ nanorods calcined at 300 and 400 °C for 2 h

The XPS spectra in Fig. 6 are performed to investigate the chemical composite and states in 1%Au/Ce_0.75_-La_0.25_ nanorod samples calcined at 300 °C for 2 h. The XPS spectrum of Ce 3*d* shows the distinct peaks of 3*d*_3/2_ spin-orbit states and 3*d*_5/2_ spin-orbit states in Fig. 6a. As known, the peaks are located at binding energy of about 899, 903, and 916 eV normally used as the spectroscopic marker to detect the presence of Ce^4+^ state. In our case, Ce 3*d* core levels show three spin orbital doublets, which are the characteristic peaks for the tetravalent states of Ce^4+^. The peaks located at around 882.8, 888.1, and 898.4 eV are assigned to the Ce 3*d*_5/2_, and those at around 901.3, 907.0, and 916.7 eV are assigned to the Ce 3*d*_3/2_, corresponding to spin-orbit split doublets of Ce (IV) compounds. The observed results are matched with reported literatures generally [19, 28, 29, 32]. It is obvious that the samples are in the state of Ce^4+^ without any impurity of the Ce^3+^ state. Figure 6b shows the XPS spectra of the La 3*d* region of 1%Au/Ce_0.75_-La_0.25_ nanorod samples. Both the spin-orbit split 3*d*_5/2_ and 3*d*_3/2_ levels showed double-peak structures. The spin-orbit splitting between the 3*d*_3/2_ and 3*d*_5/2_ levels was about 17.0 eV, and the separation between the satellite and main peak was 4.1 eV, which agreed with reported values for La^3+^ compounds [11, 47]. As would be expected, La exits in the + 3 oxidation state and may have an important influence on the catalytic activity. The O 1*s* XPS spectrum (Fig. 6c) is asymmetric and deconvoluted into 529.3, 531.6, and 527.6 eV, respectively. The peak at 529.3 eV is assigned to lattice oxygen and that at around 531.9 eV is assigned to hydroxyl groups on the surface of the support [27, 28, 32]. The small shoulder peak at 527.6 eV is attributed to La-O, which could also reveal the presence of LaO_*x*_ in the catalysts [11, 48]. Clearly there are large numbers of hydroxyl groups on the surface of the support according to the high peak intensity. The XPS spectra in the Au 4*f* region of the catalysts calcined at 300 and 400 °C are shown in Fig. 6d. In Fig. 6d, the catalysts calcined at 300 °C showed the Au 4*f*_7/2_ binding energies signals at 84.6 eV. The signals were characteristic for cationic Au^+^ species [14, 15, 31]. In comparison, after the catalysts calcined at 400 °C, the Au 4*f*_7/2_ peak was located at binding energy of 83.6 eV, and Au 4*f*_*5/2*_ was located at binding energy of 87.7 eV. The presence of metallic Au^0^ is clearly observed. The small peaks located around 85.0 and 88.2 eV, corresponding to oxidized gold species, was also detected. It is clearly that the catalysts calcined at 300 °C showed practically mainly cationic Au^+^ species (> 90% of Au^+^ species). In contrast, the samples calcined at 400 °C have 90% of Au^0^ and 10% of Au^δ+^. The electron density transfer from metallic Au towards the support resulted in the partial oxidation of Au and strong interaction between gold and support. The presence of Au^δ+^ is responsible for the partial reduction of the support surface. Accordingly, Au^δ+^ is considered to be more active than Au^0^ for CO oxidation [11, 21]. In our case, the catalysts calcined at 300 °C had more Au^δ+^ than that calcined at 400 °C, so it is not difficult to deduce the catalysts calcined at 300 °C were more active than the catalysts calcined at 400 °C, which was consistent with the activity results.

#### Uv-visible

**Fig. 7:**
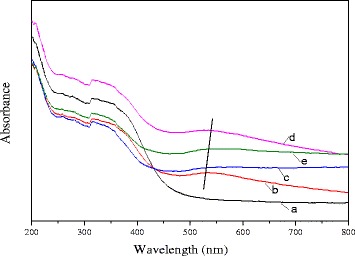
UV-vis DRS of pure Ce_0.75_-La_0.25_ nanorod support (a) and 0.3% Au/Ce_0.75_-La_0.25_ nanorods calcined at 80 °C (b), 200 °C (c), 300 °C (d), and 400 °C (e)

The UV-vis diffuse reflectance spectrum of the Ce_0.75_-La_0.25_ nanorods and 0.5% Au/Ce_0.75_-La_0.25_ nanorods calcined at different temperatures are presented in Fig. 7. As seen in this figure, compared with the spectra of the support, the spectra of the catalysts calcined at different temperatures exhibited weak and broad absorption band between 500 and 600 nm which was characterize for the surface plasmon resonance (SPR) of metallic gold nanoparticles [21, 24, 49]. The SPR could be ascribed to the collective oscillations of electrons in response to optical excitation, which would result in the absorption of light in the Uv-vis region. The location of the surface plasmon resonance was affected by the dispersed gold particle size, the shape of the particle, and the dielectric properties of the surrounding material. In the present study, the calcination pretreatment caused a large red shift of the absorption bands, and the positions of the absorption bands (500–600 nm) were red-shifted upon calcination temperature increasing. The shift ranks are as follows: 80 °C→200 °C→300 °C. With a further increase of calcination temperature to 400 °C, the absorption bands moved to the short wavelength. There were several reports about the explanation of shifting peak position [24, 50–53]. While the diameter of gold particle is < 2 nm, the broadened shifting peak position was mainly caused by size-dependent damping of the metal dielectric function. There also exited a reduction of electron density in the gold particles owing to chemical interactions with the surrounding metal oxides, which could explain the mechanism leading to a red shift further [52]. An increase in the size of the gold particles would cause a blue shift in the absorption peak (mean diameter smaller than 25 nm), and for large particles (mean diameter larger than 25 nm), the opposite effect was observed [53]. According to the TEM data, the size of the gold particles in the catalyst was < 1 nm for catalyst calcined at 300 °C. However, with a further increase of calcination temperature to 400 °C, the gold particles grew, and average size was about 7 nm. As mentioned before, the position of plasmon band strongly depended on the shape and the size of the gold particles. In the present case, this large shift might be explained in terms of the difference in the size of the gold particles. The data was consistent with the results of catalytic activity test. It also indicated that gold nanoparticles dispersed well on the surface of the supports.

#### H_2_-TPR

**Fig. 8:**
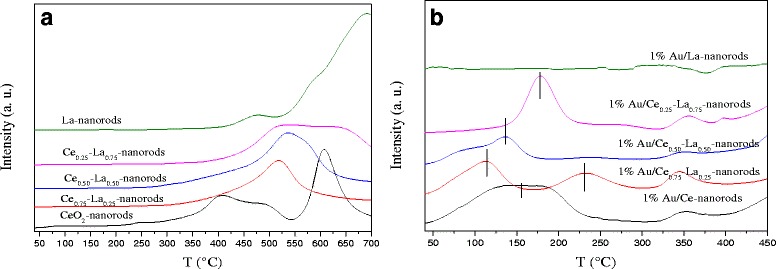
H_2_-TPR profiles of **a** La-Ce nanorods with different La content (0–100 at.%) and **b** 1% Au/La-Ce nanorods with different La content calcined at 300 °C

Figure 8a shows the TPR profile for pure and mixed oxide samples. For pure CeO_2_ nanorods, the reduction peak centered at about low temperature (410 °C) and high temperature (620 °C) could be attributed to the reduction of surface and bulk oxygen species of CeO_2_, respectively [1, 32]. For pure La nanorods, obvious reduction peaks could be detected at ~ 700 °C assigned to the reduction of bulk La_2_O_3_. It was interesting to find that the reduction peaks at ~ 500 °C of Ce-La nanorods appeared. The reduction peak of the three samples with 25, 50, and 75% at.% La doping shows a shift to higher temperature by about 20 °C upon La doping. When the La content is 25 at.%, a strongly reduction peak temperature of 520 °C was observed. It is a new reduction temperature and remarkably relative to that of pure CeO_2_ nanorods. In comparison with the reference, due to the synergist interaction between La–O and Ce–O, the reduction temperature of Ce-La nanorods was higher than pure CeO_2_ [31, 54]. It could be found that the binary oxides should have independent CeO_2_ and LaO_*x*_. As shown in Fig. 8b, after the deposition of gold, a new reduction peak at very low temperature (100–200 °C) appears for Au/CeO_2_ and Au/La-Ce nanorods. Here, due to XPS results, after the catalysts were calcined at 300 °C, Au was mainly Au^δ+^, so the reduction peaks at ~ 200 °C is attributed to the reduction of Au species in high valence [21]. The small peak centered at ~ 350 °C can be associated with the reduction of Ce-La nanorods. In addition, for 1% Au/Ce_0.75_-La_0.25_ nanorods, another reduction peak at around 230 °C can be ascribed to the gold promoted reduction of CeO_2_. One percent Au/Ce_0.75_-La_0.25_ nanorods has the lowest reduction temperature among the catalysts, which could help it being the most active catalyst for CO oxidation. This was in agreement with the activity results. Since the surface reduction peaks for all oxide supports are significantly decreased after gold deposition, it indicates that most available oxygen is reduced at this lower temperature and suggests that H_2_ dissociation on gold and spill-over onto the adjacent oxide surface are more likely to be responsible for the strong low-temperature reduction peak [31]. TEM and XPS data indicated that the cationic gold particles with small size highly dispersed on the surface of the supports. The presence of LaO_*x*_ could also help stabilize cationic Au. This is beneficial for the strength of gold-support interaction [11]. The strong interaction between gold and support promoted the reduction of Au/Ce-La nanorods shifted to low temperature. The results indicated that the reducibility of Ce-La nanorods is strongly affected by the gold deposition.

### Catalytic Activity

#### Effect of La Content

**Fig. 9:**
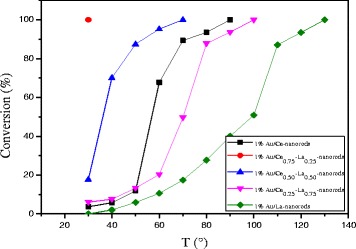
Catalytic activities of 1% Au/ La-Ce nanorods with different La content calcined at 300 °C for 2 h

As shown in Fig. 9, catalytic activity results for Au/Ce-La nanorod samples ranging from pure CeO_2_ to 100 at.% La-content nanorod supports. The most striking feature in the figure is the high activity of the Au/Ce_0.75_-La_0.25_ nanorod catalyst with the 100% conversion at temperature as low as 30 °C. In contrast, the other Au/La-Ce catalysts showed lower activity compared to Au/Ce_0.75_-La_0.25_ nanorods catalysts under the same reaction conditions. The results indicated that La doping has very much impact on this high CO conversion activity with a La content of 25 at.%, while a further increase results in a significant drop in activity. This again closely mirrors the trends seen in the reducibility of the samples, where an increase of La content from 25 at.% results in a strong loss of reducibility.

In consideration of the preparation methods, gold loadings, gold particle size and distribution on different Ce-La nanorods supports, XRD, TEM and XPS data showed that all the catalysts should have the same number and type of active Au sites. So this high activity of the Au/Ce_0.75_-La_0.25_ nanorods catalysts correlates well with the reducibility data discussed above. H_2_-TPR results indicated that Au/Ce_0.75_-La_0.25_ nanorods has the lowest reducibility temperature and highest reducibility in the region of 50–400 °C, especially in the region of 50–150 °C, which could exactly approach the region of reaction temperature. In the process of reaction, the Ce_0.75_-La_0.25_ nanorod support served as oxygen carrier. The reducibility of Ce_0.75_-La_0.25_ nanorods could promote the formation of active oxygen. That is to say high reducibility of the catalyst, good activity the catalyst has. Au/Ce_0.75_-La_0.25_ nanorod catalyst subsequently has the best activity.

#### Effect of Gold Content

**Fig. 10:**
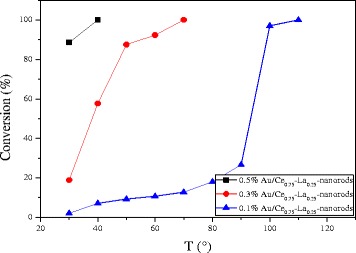
Catalytic activities of Au/Ce_0.75_-La_0.25_ nanorods with different gold goadings calcined at 300 °C for 2 h

The catalytic activities for CO oxidation were measured from low conversion to 100% conversion for the Au/Ce_0.75_-La_0.25_ nanorod catalysts calcined at 300 °C for 2 h with a series of low gold contents. As shown in Fig. 10, all of the catalysts showed high catalytic activities. The CO conversion increased greatly with increasing gold content. The complete CO conversion could be attained at 50 °C over 0.5% Au/Ce_0.75_-La_0.25_ nanorod catalyst. The size and chemical states of gold nanoparticles are generally thought to be vital for the performance of supported gold catalysts. It has been reported that its gold nanoparticles with the diameter of < 5 nm would be suitable for the supported gold catalysts in the catalytic CO oxidation [27, 28]. The XPS data proved that gold in Au/Ce_0.75_-La_0.25_ nanorod catalyst exists in the form of cationic Au^+^. TEM images of the samples were also shown to investigate the diameter of gold nanoparticles in the catalysts. Consequently, the gold particles of Au/Ce_0.75_-La_0.25_ nanorods were detected as sub-nanometer. Taking into account the particle size, mass content, and chemical states of the gold nanoparticles, gold particles with small diameter highly dispersed on the surface of Ce_0.75_-La_0.25_ nanorods and interacted strongly with the support [17, 21, 23]. The strong interaction between gold particles and the support would help improve CO adsorption and accelerate active oxygen spillover to gold particles from the support, so 0.5% Au/Ce_0.75_-La_0.25_ nanorods which had relatively high content of gold should exhibit the best CO oxidation activity. In fact, 0.5% Au/Ce_0.75_-La_0.25_ nanorods indeed present high performance. The results demonstrated the activity of supported gold catalysts is strongly dependent on the gold nanoparticle size, chemical states, and the quantity of the active species, an increase of which implied an increase of the catalytic activity. In the case of Au/Ce_0.75_-La_0.25_ nanorod catalyst, catalysts with low gold content could also exhibits high activity at low temperature, which would promote the progress of supported gold catalyst. The results indicated that supported gold catalysts prepared by deposition-precipitation with pH value of 6–10 for HAuCl_4_ solution could have high catalytic activity due to small diameter of gold nanoparticles, corresponding with the references [8–10].

#### Effect of Calcination Temperature

**Fig. 11:**
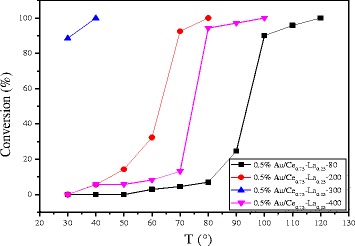
Catalytic activities of 0.5% Au/Ce_0.75_-La_0.25_ nanorods calcined at different temperatures

The effect of calcination temperature on the catalytic activity of 0.5%Au/Ce_0.75_-La_0.25_ nanorods is also shown in Fig. 11. The results indicated an increase in the activity of catalyst with the calcination temperature from 80 to 300 °C. The 0.5% Au/Ce_0.75_-La_0.25_ nanorod catalyst calcined at 200 °C could convert CO to CO_2_ completely at 80 °C. While for 0.5% Au/Ce_0.75_-La_0.25_ nanorod catalyst calcined at 80 °C, the temperature increased to 100 °C. The results showed that CO conversion increased with increasing calcinations temperature. Then, for the sample calcined at 400 °C, about 90% CO can be converted to CO_2_ at 100 °C and CO could be converted to CO_2_ completely at 120 °C. The sample calcined at 300 °C possessed the best catalytic activity. The catalytic performance of supported gold catalysts strongly depends on gold nanoparticle size and metal-support interaction due to “synergic effect” at the gold-support interface [10, 13, 15, 18]. The gold-support interaction largely depended on calcination temperature of catalysts. The electron could transfer from gold to the support [10]. Thus, with increasing calcination temperature, the charges on gold particles became increasingly positive, which is good for the enhancement of catalytic activity for CO oxidation. Here, as shown in the Fig. 5, size of gold particles in the catalysts calcined at 300 °C was small. The XPS data also indicated that gold was main Au^δ+^ after calcination at 300 °C. Thus, the stronger metal-support interaction could account for the relative good catalytic performance for catalysts calcined at 300 °C. From 80 to 300 °C, the higher the calcination temperature is, the stronger interaction exists between gold particles and support. As a consequence, from 80 to 300 °C, the activity of catalysts increased. However, after the 0.5% Au/Ce_0.75_-La_0.25_ nanorod catalyst calcined at 400 °C, complete conversion temperature increased. The main reason might be that the high-temperature treatment led to increased mobility and growth of gold nanoparticles, which correspondingly led to the loss of catalytic activity. The XPS also suggested that the catalysts calcined at 400 °C, Au was mainly Au^0^. It could be concluded that the activities of supported gold nanoparticles were influenced by both the oxidation state and the size of gold nanoparticles, and the appropriate calcination temperature was 300 °C.

#### Stability Observations

**Fig. 12:**
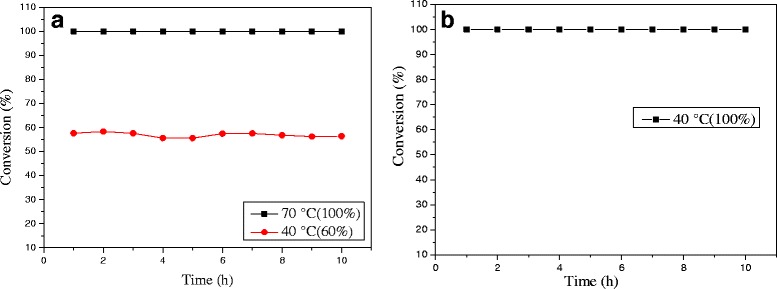
The stability of 0.3% Au/Ce_0.75_-La_0.25_ nanorods, reaction temperature: 40 and 70 °C (**a**) and 0.5% Au/Ce_0.75_-La_0.25_ nanorods, reaction temperature: 40 °C (**b**) for the CO oxidation

The stability of the 0.3% Au/Ce_0.75_-La_0.25_ nanorod catalyst during CO oxidation at different reaction temperatures was measured, as shown in Fig. 12a. When the reaction was carried out at 70 °C, the initial CO conversion over 0.3% Au/Ce-La catalyst can reach 100% and has almost no change with continuously increasing reaction time. 0.3% Au/Ce-La catalyst with 60% CO conversion rate at 40 °C is also attained even after 10-h running period, and no obvious decline in CO conversion is observed. Although the catalytic activity of 0.3% Au/Ce_0.75_-La_0.25_ nanorod catalyst at 40 °C was lower than that of 0.3% Au/Ce_0.75_-La_0.25_ nanorod catalyst at 70 °C, the conversion of CO over the catalysts at both temperatures still seemed to be stable over 10 h on stream. It is thought that the catalyst was of good durability. It was clear that the activity over 0.3% Au/Ce_0.75_-La_0.25_ nanorod catalyst did not strongly depend on the reaction temperature. As the reaction temperature decrease the activation rate barely becomes little slower and then finally reaching a steady state in which the CO conversion was still around 90%. For comparison, the stability of 0.5% Au/Ce_0.75_-La_0.25_ nanorod catalyst at the reaction temperature of 40 °C with initial conversion of 100% was also provided in Fig. 12b. It was obvious that in 10 h, no decrease of CO conversion for 0.5% Au/Ce_0.75_-La_0.25_ nanorods was detected. The results depicted that with the change of gold content, Au/Ce_0.75_-La_0.25_ nanorods could still perform good stability.

**Fig. 13:**
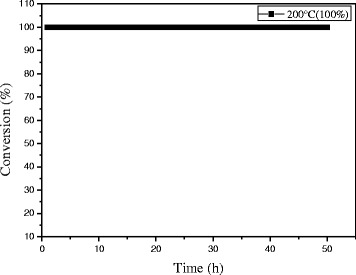
The stability of 0.3% Au/Ce_0.75_-La_0.25_ nanorods at the reaction temperature of 200 °C for CO oxidation

As engine efficiency increases and automotive exhaust temperatures decrease, traditional supported gold catalysts would be insufficient to meet emission regulations. And there are also a number of industrial catalytic processes which (e. g., the catalytic oxidation of CO in automotive exhaust gas) are sometimes carried out at high temperatures. Thus, the development of new catalysts that are active at lower temperature, yet still stable at periodic high temperatures, will be vital. In the two regards, catalysts with good activity at low temperature that are stable at high reaction temperatures are desirable. It is necessary to investigate their catalytic performance for CO oxidation at a certain high temperature which is a very stringent test for the stability of gold nanocatalysts against sintering. In the present work, the stability of 0.3% Au/Ce_0.75_-La_0.25_ nanorod catalyst was also measured at 200 °C (100%) for high-temperature treatment. As shown in Fig. 13, no decline of catalytic activity was observed within 50 h indicates that the catalyst keeps good stability within 50 h. Remarkably, very few serious gold sintering occurred during the reaction. It indicated that 0.3% Au/Ce-La catalyst can exhibit good catalytic stability at both low and high reaction temperatures.

#### Reaction Mechanism Speculate

**Fig. 14:**
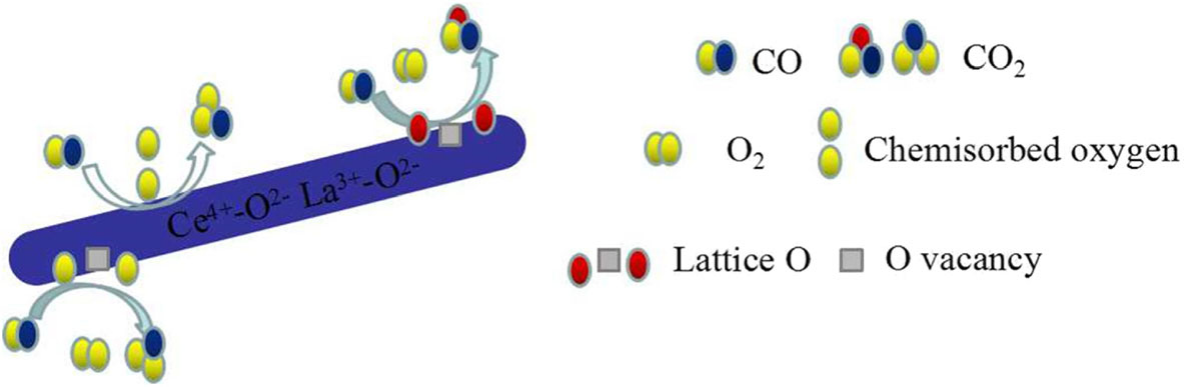
Proposed CO reaction pathways over the catalysts, Au/Ce-La nanorods

Combined with H_2_-TPR and XPS experiments, it suggested that CO oxidation over LaO_*x*_-doped CeO_2_-supported Au catalysts might follow the Langmuir–Hinshelwood + Redox mechanism [1, 20, 26, 30, 32]. The XPS results suggest that there are Ce^3+^ and Ce^4+^ on the surface of the catalyst. H_2_-TPR data also proved that reducibility of this binary Ce-La nanorod oxides could be promoted by Au deposition. The reducibility of Au/Ce-La nanorods was much higher than pure Au/CeO_2_ or Au/LaO_*x*_ catalysts. This would help the produce of oxygen vacancies. The oxygen vacancy is a very lively activity center. The active center can promote the activation of O_2_. Thus, the CO oxidation reaction could become more easily. There are also amount of adsorbed oxygen species on the surface of catalyst. Usually adsorbed oxygen species play an important role in the oxidation of CO. The O_2_ of the reaction will form the chemisorbed oxygen, and the oxygen vacancy would be replenished by O_2_ of reaction gas to form new active lattice oxygen. XPS data also proved that gold in the catalysts was mainly Au^δ+^ species, which would accelerate the adsorption of CO. The possible reaction mechanisms of Au/Ce-La nanorod catalyst could be described as follows. Firstly, CO and O_2_ were chemisorbed on the surface of the catalysts. Then, the chemisorbed oxygen directly reacts with CO, or the active lattice oxygen of the catalyst reacts with CO, and the catalyst produced the oxygen vacancy with oxygen from gas-phase O_2_. At last, CO was oxidized into CO_2_ (shown in Fig. 14).

## Conclusions

In summary, a series of mixed Ce-La nanorods with various amounts of La was prepared via a simple hydrothermal reaction at high concentration of NaOH and without surfactant. Gold was loaded by deposition-precipitation. After La doping, the composite could retain the initial rod morphology. As a result, Ce-La nanorods with 25 at.% La maintained the optimal nanorods with the length of 0.6 um and the diameter of 3–5 nm. Gold particles were dispersed well on the support. The reducibility of Ce-La nanorods could be affected significantly by LaO_*x*_ doping. The deposition of gold had important influence on the reducibility of catalyst. Thus, the CO oxidation activity of Au/Ce-La nanorods was essentially changed in comparison with pure Au/CeO_2_ and Au/La nanorods. One percent Au/Ce_0.75_-La_0.25_ nanorods could convert CO to CO_2_ completely at 30 °C. Further increase in La content results in decreased activity due to the decrease in reducible oxygen sites. The Au/Ce_0.75_-La_0.25_ nanorod catalyst with low gold concentration also showed high activity. With the increase of gold content, there is an increase in the activity. The stability test of 0.3% Au/Ce_0.75_-La_0.25_ nanorods indicated that the catalyst not only kept 100% conversion after continuous operation for 10 h under 70 °C but also showed no deactivation after 10 h on stream at 40 °C. As expected, the activity of 0.3% Au/Ce_0.75_-La_0.25_ nanorods also retained a 100% CO conversion during 50 h at 200 °C. The results revealed that LaO_*x*_ as the dopant could grow together with CeO_2_ in one rod. The 1D binary mixed Ce-La nanorods could be a good support for precious metal group catalysts with low content of gold.

## References

[1] Zheng Y, Li KZ, Wang H, Wang YH, Tian D, Wei YG, Zhu X, Zeng CH, Luo YM (2016). Structure dependence and reaction mechanism of CO oxidation: a model study on macroporous CeO_2_ and CeO_2_-ZrO_2_ catalysts. J Catal.

[2] JM W, Zeng L, Cheng DG, Chen FQ, Zhan XL, Gong JL (2016). Synthesis of Pd nanoparticles supported on CeO_2_ nanotubes for CO oxidation at low temperatures. Chin J Catal.

[3] He HY, Yang P, Li J, Shi RX, Chen L, Zhang AY, Zhu YN (2016). Controllable synthesis, characterization, and CO oxidation activity of CeO_2_ nanostructures with various morphologies. Ceram Int.

[4] Gao JJ, Jia CM, Zhang LP, Wang HM, Yang YH, Hung SF, Hsu YY, Liu B (2016). Tuning chemical bonding of MnO_2_ through transition-metal doping for enhanced CO oxidation. J Catal.

[5] Wang H, Zhu HQ, Qin ZF, Liang FX, Wang GF, Wang JG (2009). Deactivation of a Au/CeO_2_-Co_3_O_4_ catalyst during CO preferential oxidation in H_2_-rich stream. J Catal.

[6] Soler L, Casanovas A, Urrich A, Angurell I, Llorca J (2016). CO oxidation and COPrOx over preformed Au nanoparticles supported over nanoshaped CeO_2_. Appl Catal B.

[7] Zhang XM, Deng YQ, Tian PF, Shang HH, Xu J, Han YF (2016). Dynamic active sites over binary oxide catalysts: in situ/operando spectroscopic study of low-temperature CO oxidation over MnO_x_-CeO_2_ catalysts. Appl Catal B.

[8] Zhang S, Li XS, Zhu B, Liu JL, Zhu XB, Zhu AM, Jang BWL (2015). Atmospheric-pressure O_2_ plasma treatment of Au/TiO_2_ catalysts for CO oxidation. Catal Today.

[9] Haruta M (1997). Size- and support-dependency in the catalysis of gold. Catal Today.

[10] Liu XY, Liu MH, Luo YC, Mou CY, Lin SD, Cheng HK, Chen JM, Lee JF, Lin TS (2012). Strong metal-support interactions between gold nanoparticles and ZnO nanorods in CO oxidation. Int J Hydrog Energy.

[11] Clarka PD, Sui R, Dowling NI, Huang M, Lo JMH (2013). Oxidation of CO in the presence of SO_2_ using gold supported on La_2_O_3_/TiO_2_ nanofibers. Catal Today.

[12] Reina TR, Ivanova S, Centeno MA, Odriozola JA (2015). Catalytic screening of Au/CeO_2_-MO_x_/Al_2_O_3_ catalysts (M ¼ La, Ni, Cu, Fe, Cr, Y) in the CO-PrOx reaction. Int J Hydrog Energy.

[13] Ayastuy JL, Gurbani A, Guti errez-Ortiz MA (2016). Effect of calcination temperature on catalytic properties of Au/Fe_2_O_3_ catalysts in CO-PROX. Int J Hydrog Energy.

[14] Lee DS, Chen YW (2016). Au/CuO-CeO_2_ catalyst for preferential oxidation of CO in hydrogen-rich stream: effect of CuO content. Int J Hydrog Energy.

[15] Hernandez JA, Gomez SA, Zepeda TA, Gonzalez JCF, Fuentes GA (2015). Insight into the deactivation of Au/CeO_2_ catalysts studied by in situ spectroscopy during the CO-PROX reaction. Catalogue.

[16] Carabineiro SAC, Bogdanchikova N, Pestryakov A, Tavares PB, Fernandes LSG, Figueiredo JL (2016). Gold nanoparticles supported on magnesium oxide for CO oxidation. Nanoscale Res Lett.

[17] Mock SA, Sharp SE, Stoner TR, Radetic MJ, Zell ET, Wang RG (2016). CeO_2_ nanorods-supported transition metal catalysts for CO oxidation. J Colloid Interface Sci.

[18] Lin YY, ZL W, Wen JG, Ding KL, Yang XY, Poeppelmeier KR, Marks LD (2015). Adhesion and atomic structures of gold on ceria nanostructures: the role of surface structure and oxidation state of ceria supports. Nano Lett.

[19] He GP, Fan HQ, Ma LT, Wang KG, Liu C, Ding DH, Chen L (2016). Dumbbell-like ZnO nanoparticles-CeO_2_ nanorods composite by one-pot hydrothermal route and their electrochemical charge storage. Appl Surf Sci.

[20] Li FY, Li L, Liu XY, Zeng XC, Chen ZF (2016). High-performance Ru_1_/CeO_2_ single-atom catalyst for CO oxidation: a computational exploration. Chem Phys Chem.

[21] Cordoba LF, Hernandez AM (2015). Preferential oxidation of CO in excess of hydrogen over Au/CeO_2_-ZrO_2_ catalysts. Int J Hydrog Energy.

[22] Sudarsanam P, Hillary B, Amin MH, Abd-Hamid SB, Bhargava SK (2016). Structure-activity relationships of nanoscale MnO_x_/CeO_2_ heterostructured catalysts for selective oxidation of amines under eco-friendly conditions. Appl Catal B.

[23] Piqueras CM, Puccia V, Vega DA, Volpe MA (2016). Selective hydrogenation of cinnamaldehyde in supercritical CO_2_ over Me-CeO_2_ (Me = Cu, Pt, Au): insight of the role of Me-Ce interaction. Appl Catal B.

[24] Reina TR, Ivanova S, Centeno MA, Odriozola JA (2016). The role of Au, Cu & CeO_2_ and their interactions for an enhanced WGS performance. Appl Catal B.

[25] Gong X, Liu BC, Zhang G, GR X, Zhao T, Shi DC, Wang Q, Zhang J (2016). A mild and environmentally benign strategy towards hierarchical CeO_2_/Au nanoparticle assemblies with crystal facet-enhanced catalytic effects for benzyl alcohol aerobic oxidation. CrystEngComm.

[26] Bensaid S, Piumetti M, Novara C, Giorgis F, Chiodoni A, Russo N, Fino D (2016). Catalytic oxidation of CO and soot over Ce-Zr-Pr mixed oxides synthesized in a multi-inlet vortex reactor: effect of structural defects on the catalytic activity. Nanoscale Res Lett.

[27] Jardim ED-O, Francés SR, Coloma F, Fernández EVR, Albero JS, Escribano AS (2014). Superior performance of gold supported on doped CeO_2_ catalysts for the preferential CO oxidation (PROX). Appl Catal A.

[28] López JM, Arenal R, Puértolas B, Mayoral Á, Taylor SH, Solsona B, García T (2014). Au deposited on CeO_2_ prepared by a nanocasting route: a high activity catalyst for CO oxidation. Catalogue.

[29] Moemen AA, Mageed AMA, Bansmann J, Wojtan MP, Behm RJ, Kučerová G (2016). Deactivation of Au/CeO_2_ catalysts during CO oxidation: influence of pretreatment and reaction conditions. Catalogue.

[30] Good J, Duchesne PN, Zhang P, Koshut W, Zhou M, Jin RC (2017). On the functional role of the cerium oxide support in the Au_38_(SR)_24_/CeO_2_ catalyst for CO oxidation. Catal Today.

[31] Luengnaruemitchai A, Chawla S, Wanchanthuek R (2014). The catalytic performance of Au/La-CeO_x_ catalyst for PROX reaction in H_2_ rich stream. Int J Hydrog Energy.

[32] Piumetti M, Andana T, Bensaid S, Russo N, Fino D, Pirone R (2016). Study on the CO oxidation over ceria-based nanocatalysts. Nanoscale Res Lett.

[33] Huang XS, Sun H, Wang LC, Liu YM, Fan KN, Cao Y (2009). Morphology effects of nanoscale ceria on the activity of Au/CeO_2_ catalysts for low-temperature CO oxidation. Appl Catal B.

[34] Ma TY, Yuan ZY, Cao JL (2010) Hydrangea-Like Meso−/Macroporous ZnO-CeO2 binary oxide materials: synthesis, Photocatalysis and CO oxidation. Eur J Inorg Chem 716-724

[35] Bao HZ, Chen X, Fang J, Jiang ZQ, Huang WX (2008). Structure-activity relation of Fe_2_O_3_-CeO_2_ composite catalysts in CO oxidation. Catal Lett.

[36] Tsoncheva T, Ivanova R, Henych J, Velinov N, Kormunda M, Dimitrov M, Paneva D, Slušná M, Mitov I, Štengl V (2016). Iron modified titanium–hafnium binary oxides as catalysts in total oxidation of ethyl acetate. Catal Commun.

[37] Ma Z, Overbury SH, Dai S (2007). Au/M_*x*_O_*y*_/TiO_2_ catalysts for CO oxidation: promotional effect of main-group, transition, and rare-earth metal oxide additives. J Mol Catal A Chem.

[38] Park JB, Graciani J, Evans J, Stacchiola D, Senanayake SD, Barrio L, Liu P, Sanz JF, Hrbek J, Rodriguez JA (2010). Gold, copper, and platinum nanoparticles dispersed on CeO_*x*_/TiO_2(110)_ surfaces: high water-gas shift activity and the nature of the mixed-metal oxide at the nanometer level. J Am Chem Soc.

[39] Río ED, Hungría AB, Tinoco M, Manzorro R, Cauqui MA, Calvino JJ, Omil JAP (2016). CeO_2_-modified Au/TiO_2_ catalysts with outstanding stability under harsh CO oxidation conditions. Appl Catal B.

[40] Sahu N, Parida KM, Tripathi AK, Kamble VS (2011). Low temperature CO adsorption and oxidation over Au/rare earth-TiO_2_ nanocatalysts. Appl Catal A.

[41] Yu P, Wu J, Liu ST, Xiong J, Jagadish C, Wang ZM (2016). Design and fabrication of silicon nanowires towards efficient solar cells. Nano Today.

[42] Zhu Y, Appenzeller J (2015). On the current drive capability of low dimensional semiconductors: 1D versus 2D. Nanoscale Res Lett.

[43] Kang JG, Kim YI, Cho DW, Sohn Y (2015). Synthesis and physicochemical properties of La(OH)_3_ and La_2_O_3_ nanostructures. Mater Sci Semicond Process.

[44] Yi N, Si R, Saltsburg H, Stephanopoulos MF (2010). Steam reforming of methanol over ceria and gold-ceria nanoshapes. Appl Catal B.

[45] Tabakova T, Boccuzzi F, Manzoli M, Scobczak JW, Idakiev V, Andreeva D (2006). A comparative study of nanosized IB/ceria catalysts for low-temperature water-gas shift reaction. Appl Catal A.

[46] Yi N, Si R, Saltburg H, Stephanopoulos MF (2010). Active gold species on cerium oxide nanoshapes for methanol steam reforming and the water gas shift reactions. Energy Environ Sci.

[47] Majumdar S, Kooser K, Elovaara T, Huhtinen H, Granroth S, Paturi P (2013). Active gold species on cerium oxide nanoshapes for methanol steam reforming and the water gas shift reactions. J Phys Condens Matter.

[48] Pawlak DA, Ito M, Oku M, Shimamura K, Fukuda T (2002). Interpretation of XPS O (1s) in mixed oxides proved on mixed perovskite crystals. J Phys Chem B.

[49] Reina TR, Ivanova S, Idakiev V, Tabakova T, Centeno MA, Deng QF, Yuan ZY, Odriozola JA (2016). Nanogold mesoporous iron promoted ceria catalysts for total and preferential CO oxidation reactions. J Mol Catal A.

[50] Kelly KL, Coronado E, Zhao LL, Schatz GC (2003). The optical properties of metal nanoparticles: the influence of size, shape, and dielectric environment. J Phys Chem B.

[51] Westcott SL, Oldenburg SJ, Lee TR, Halas NJ (1998). Formation and adsorption of clusters of gold nanoparticles onto functionalized silica nanoparticle surfaces. Langmuir.

[52] Carrot G, Valmalette JC, Plummer CJG, Scholz SM, Dutta J, Hofmann H, Hilborn JG (1998). Gold nanoparticle synthesis in graft copolymer micelles. Colloid Polym Sci.

[53] Link S, El-Sayed MA (1999). Size and temperature dependence of the plasmon absorption of colloidal gold nanoparticles. J Phys Chem B.

[54] Liang S, Broitman E, Wang YN, Cao AM, Veser G (2011). Highly stable, mesoporous mixed lanthanum–cerium oxides with tailored structure and reducibility. J Mater Sci.

